# Supplementary material for: Neural architecture and regeneration in the acoel Hofstenia miamia
Source: Proc Biol Sci. 2020 Jul 22;287(1931):20201198. doi: 10.1098/rspb.2020.1198 (PMC7423668; doi:10.1098/rspb.2020.1198)

Supplementary Method and Table, Figure, and Movie Legends

Supplementary Method: Immunohistochemistry for FMRFamide, Tyrosinated tubulin, and Tropomyosin antibodies

Animals were fixed in 4% paraformaldehyde in artificial sea water for 30 minutes at room temperature and subsequently moved to PBST. Animals were then permeabilized in 100% methanol for 10 minutes and moved back to PBST. Block solution (10% Horse Serum in PBST) was applied for 30 minutes. Primary antibodies (FMRFamide; EMD Millipore AB15348, Tyrosinated tubulin; Sigma-Aldrich T9028) were diluted in block solution at 1:300 (Tropomyosin antibodies used together at a final concentration of 1 $\mu g\ ml^{-1}$) and incubated for 72 hours at 4°C. Specimens were washed 2x10 minutes and 2x30 minutes in PBST and incubated for 24 hours at 4°C in secondary antibodies diluted 1:200 in block solution (Alexa Fluor 488 goat anti-rabbit; Jackson ImmunoResearch Laboratories, 111-545-003 or Alexa Fluor 568 goat anti-mouse; Abcam, 175701). Specimens were washed 2x10 minutes and 2x30 minutes in PBST. Nuclear staining was performed using DAPI® (VWR, 80051-386) (1 $\mu g\ ml^{-1}$) for 30 minutes. Specimens were mounted in VECTASHIELD® (VWR, 101098-042).

Supplementary Table 1: Neural genes with proposed homology assessment

Table with gene family, accession number, sequence, orthology assessment and method, assigned gene name, and description of FISH pattern.

Supplementary Table 2: Tropomyosin protein coding sequence

H. miamia protein sequence for Tropomyosin.

Supplementary Table 3: Regeneration measurements

Measurements of Anterior Condensation length and Body length during regeneration.

Supplementary Figure 1: Additional neurotransmitter synthesis genes and associated phylogenies

(a) Major neurotransmitter synthesis pathways are shown with enzymes highlighted in blue (orthologs encoded in the genome) and magenta (orthologs encoded in the genome and expression pattern shown). Enzymes have been identified within the *H. miamia* genome and numbers next to each enzyme represent the number of putative orthologs. Images show dorsal projection (D) on left and ventral projection (V) on right. Dashed white lines around specimens show the outline of the animal. Scale bars 200µm. (b) Maximum likelihood trees of hydroxylases and decarboxylases. Support values from 1,000 bootstrap replicates, implemented in RAxML (v8.2.4) [44] using the WAG+G model of protein evolution, shown on branches. Phylogenetic analyses support orthology of select hydroxylases and decarboxylases. Branches highlighted in red denote genes representing *H. miamia* hydroxylases and decarboxylases used for FISH (figure 4 and supplementary figure 1a).

Supplementary Figure 2: Transverse reconstruction depicts layering of muscle and neural elements

Transverse reconstruction of nervous system (*gad-1*) and musculature (Tropomyosin) depicting the layering of the peripheral muscle, Layer I neurons, body wall muscle, and Layer II neurons.

Supplementary Figure 3: *pc2* labels diverse neurons in the two layers of the anterior condensation

Top left schematic depicts a transverse section through the anterior of the animal demarcating major neural structures, including Layer I and Layer II. To the right, co-expression data are summarized for Layer I (dark grey) and Layer II (light grey) as follows: presence of cells with only neurotransmitter gene expression (green circle), only *pc2* expression (magenta circle), or double-expression (magenta+green circle); legend on left. All *pc2*$^+$ neurons, both in Layer I and Layer II, were labeled with *ache-1*, *dbhl-1*, and *hdc*. In contrast, *th*$^+$ cells and *tph-1*$^+$ cells were only present in Layer I and showed very little to no co-expression of *pc2*. White arrowheads indicate examples of double-labeled cells. Scale bars 20µm.

Supplementary Figure 4: Cells expressing *dbhl-1*, *TrpC-1*, and *pc2* regenerate robustly

(a) Ventral view of intact animal expression of *dbhl-1* and *TrpC-1* with amputation plane indicated as dashed red line. Red boxes indicate the tail fragments imaged in the regeneration time course, shown on the right. Tail fragments at various time points hours post amputation (hpa) or days post amputation (dpa). The anterior condensation, observed as concentrated expression of *dbhl-1*, was detectable 3 dpa in tail fragments (white arrowhead). Anterior condensation, observed as concentrated expression of *TrpC-1*, was detectable 4 dpa in tail fragments (yellow arrowhead). (b) A schematic of *H. miamia* in dorsal view with amputation plane indicated as dashed red line. Tail fragment denoted with red box. Anterior condensation, observed as concentrated expression of *pc2*, is visible 8dpa in tail fragments (white arrowhead). (c) Co-expression of *TrpC-1* with *gad-1* and *pc2* demonstrates the overall spatial expression with concentration in the anterior of the animal. Top middle schematic depicts a transverse section through the anterior of the animal demarcating major neural structures, including Layer I and Layer II. To the right, co-expression data are summarized for Layer I (dark grey) and Layer II (light grey) as follows: presence of cells with only neurotransmitter gene expression (green circle), only *TrpC-1* expression (magenta circle), or double-expression (magenta+green circle); legend bottom middle. All *TrpC-1*$^+$ neurons, found only in Layer II, were labeled with *gad-1* and *pc2*. *TrpC-1* labels a subset of *gad-1*$^+$ and *pc2*$^+$ neurons in anterior condensation. White arrowheads indicate examples of double-labeled cells. Dashed white lines around specimens show the outline of the animal. Scale bars: (a,b) 200µm, (c) Intact animals: 200µm, High magnification: 20µm .

Supplementary Movie 1: Layering of muscle and neural elements in the anterior of *H. miamia*

Movie generated from confocal image z-stack. Anterior portion of animal shown with muscle (Tropomyosin, green) and neurons (*gad-1*, magenta) labeled. Corresponds to figure 3*a*, *b-e*.

Supplementary Movie 2: Layering of muscle and neural elements in the posterior of *H. miamia*

Movie generated from confocal image z-stack. Posterior portion of animal shown with muscle (Tropomyosin, green) and neurons (*gad-1*, magenta) labeled. Corresponds to figure 3*a*, *f-i*.

Supplementary Figure 1

(a)

(b)

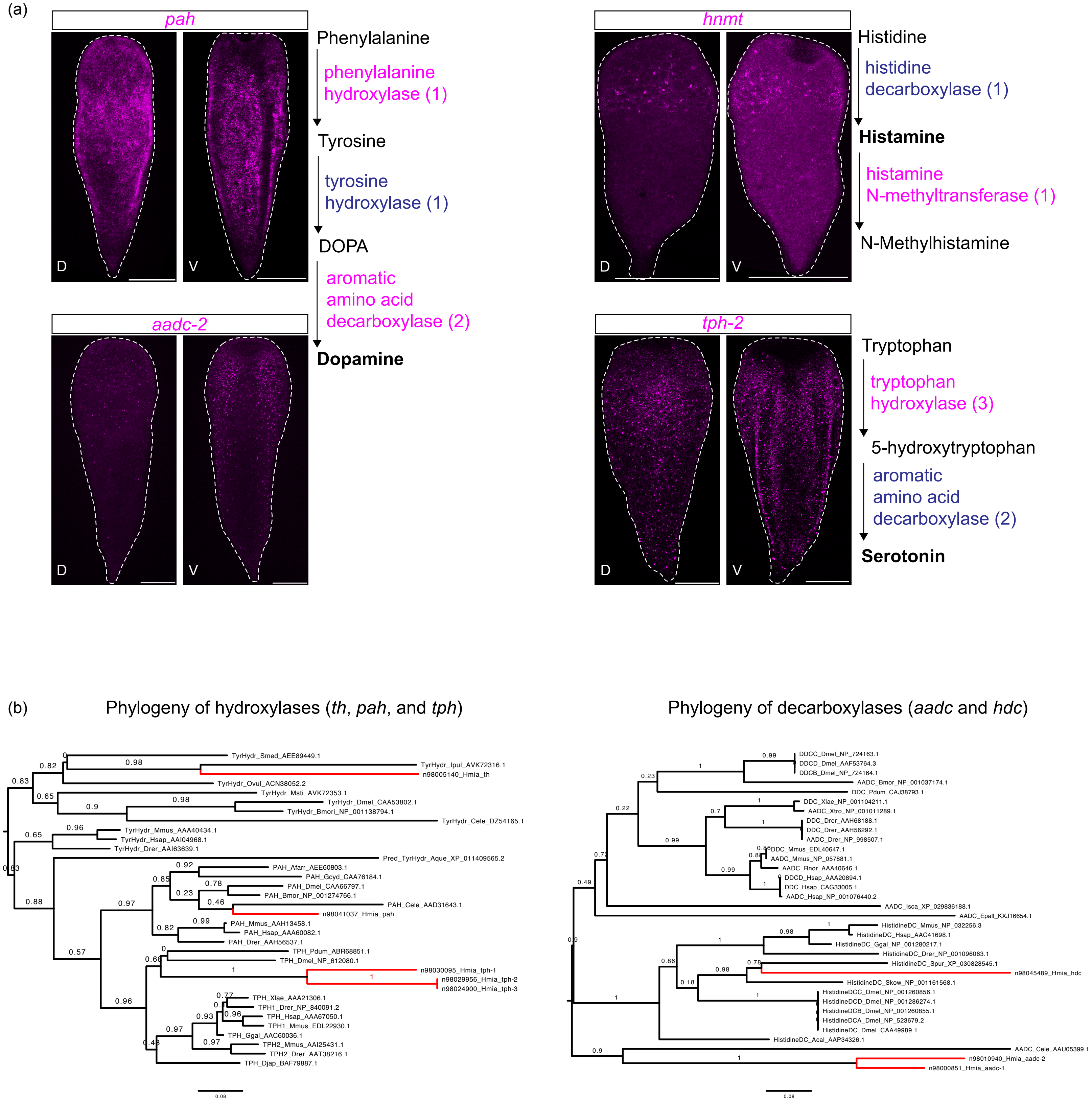

Supplementary Figure 2

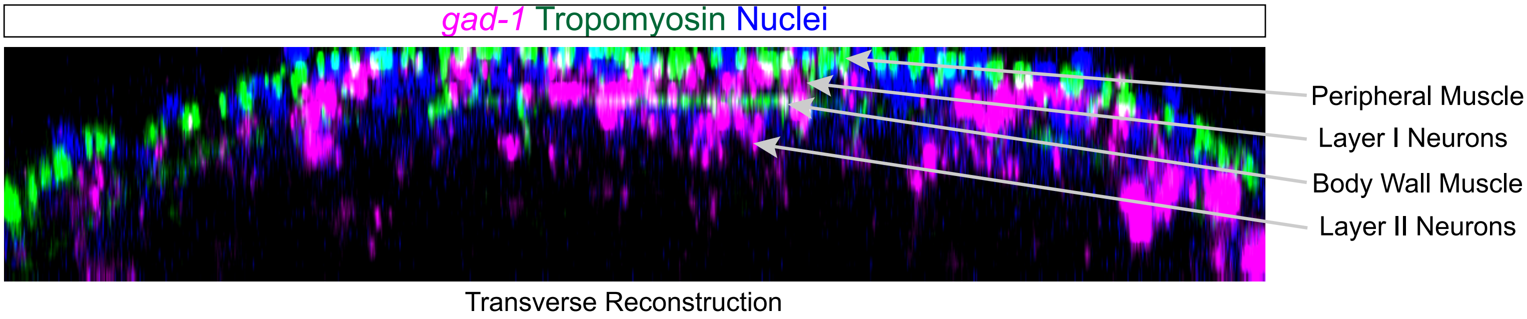

Supplementary Figure 3

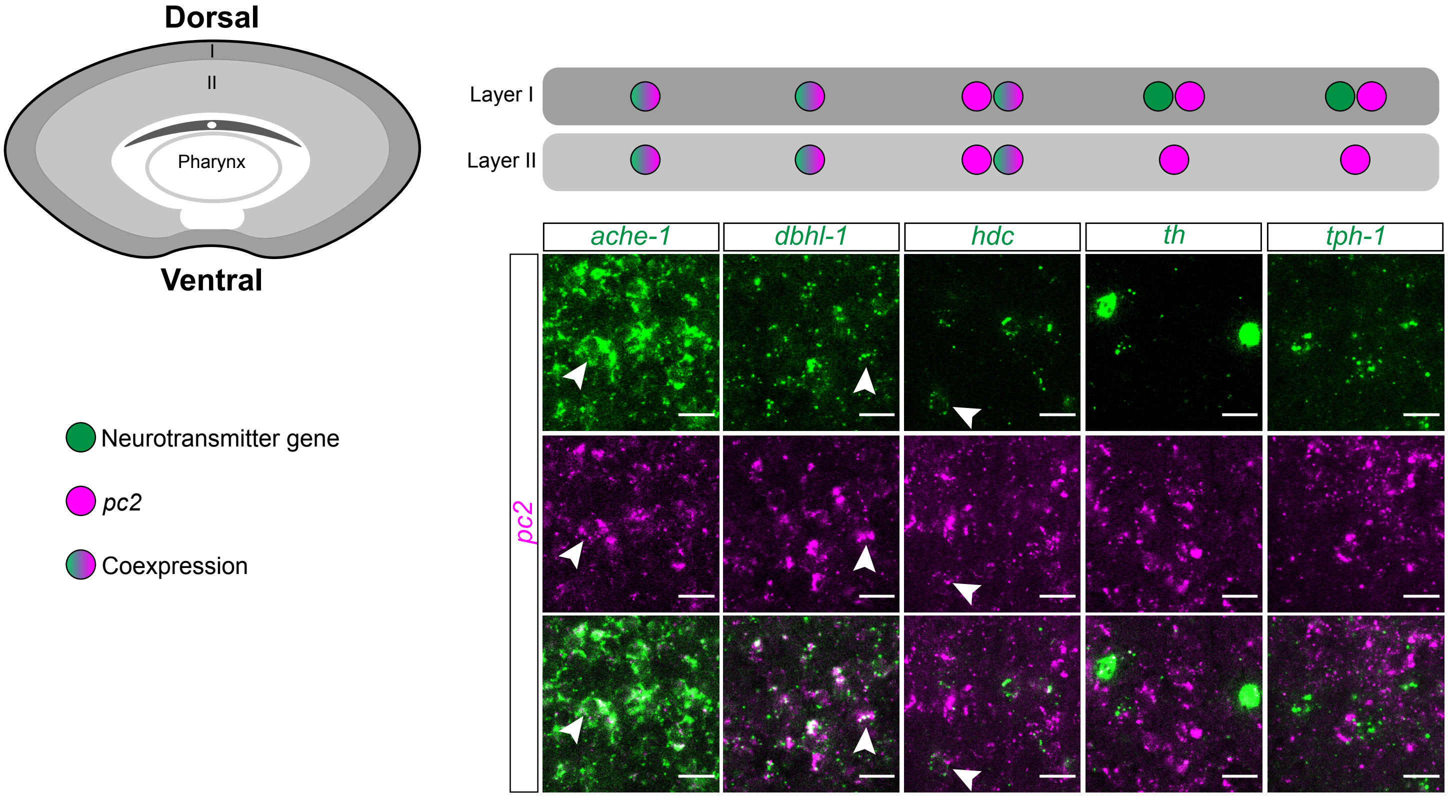

Supplementary Figure 4

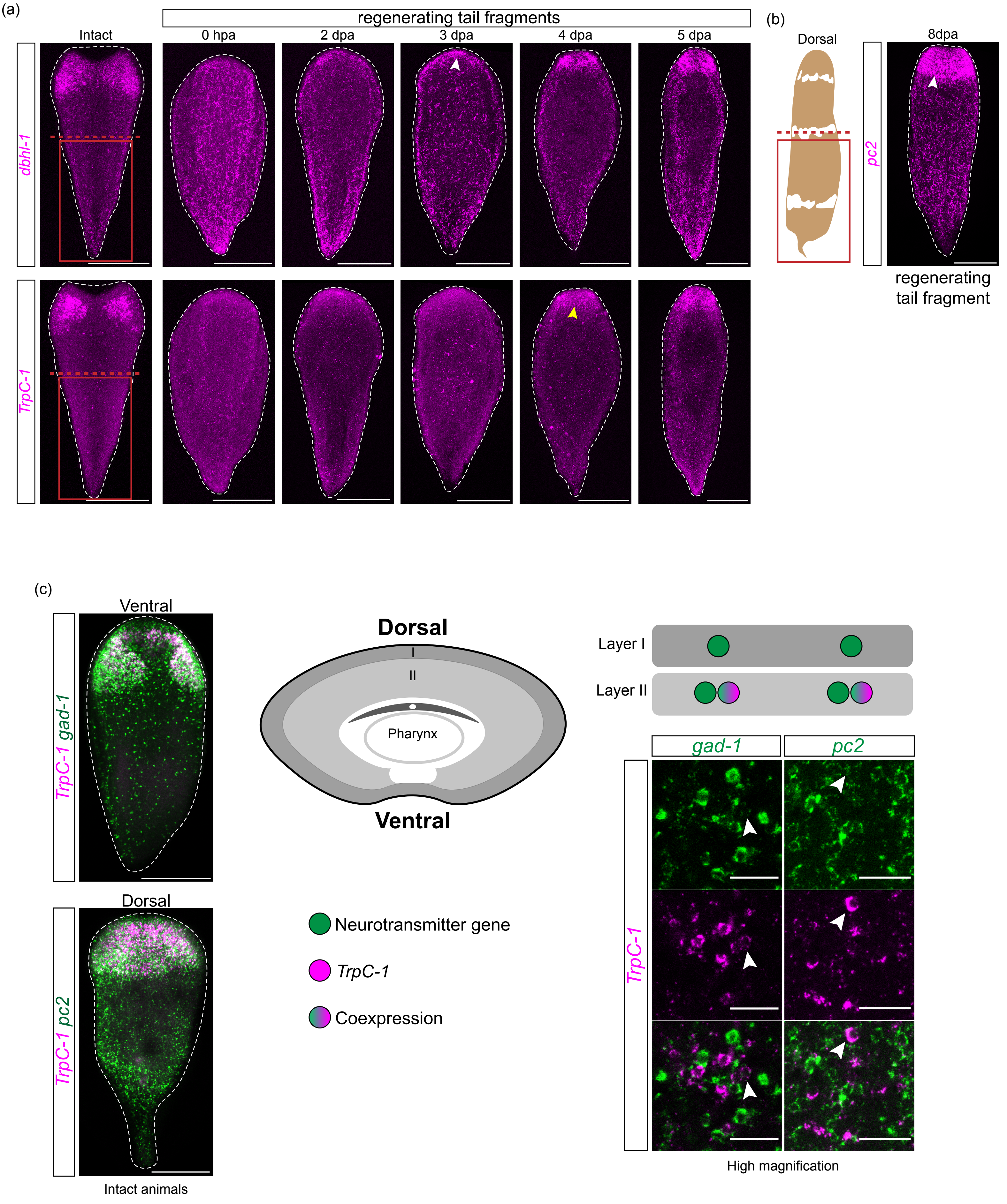

Supplement: Supplementary Figures and Legends [file rspb20201198supp1.pdf]
